# Flushing ensures vessel presealing in underwater third-space endoscopy

**DOI:** 10.1055/a-2591-1251

**Published:** 2025-06-27

**Authors:** Antonio Capogreco, Roberta Maselli, Romain Legros, Ludovico Alfarone, Cesare Hassan, Jérémie Jacques, Alessandro Repici

**Affiliations:** 1 9268 Endoscopy Unit, IRCCS Humanitas Research Hospital, Milan, Italy; 2 437807 Biomedical Sciences, Humanitas University, Milan, Italy; 3 9268 Endoscopy Unit, IRCCS Humanitas Research Hospital, Milan, Italy; 4 37925 Hepatogastroenterology, CHU Dupuytren, Limoges, France; 5 9268 Endoscopy Unit, IRCCS Humanitas Research Hospital, Milan, Italy; 6 9268 Endoscopy Unit, IRCCS Humanitas Research Hospital, Milan, Italy; 7 437807 Biomedical Sciences, Humanitas University, Milan, Italy; 8 37925 Hepatogastroenterology, CHU Dupuytren, Limoges, France; 9 9268 Endoscopy Unit, IRCCS Humanitas Research Hospital, Milan, Italy; 10 437807 Biomedical Sciences, Humanitas University, Milan, Italy

Intraprocedural bleeding due to unintentional cutting of the vessel affects both the safety and efficiency of third-space endoscopy and may be prevented by complete coagulation of the vessel before its cutting, also known as “presealing.” In a prospective series, an underwater (saline-immersion) presealing technique achieved with a large-tip knife appeared to minimize intraprocedural bleeding, saving the use and cost of coagulation forceps 1 2 . Unexpectedly, when applying the same presealing technique with a small-tip knife rather than a large-tip one, we found that the coagulation effect disappeared, with inadvertent cutting of the vessel occurring. The lack of presealing appeared to be associated with the formation of microbubbles around the tip of the knife, which was presumably owing to the different density of current for a large- and small-tip knife. In order to restore the presealing effect, we hypothesized that continuous flushing around the tip of the knife would replace the CO _2_ bubbles that formed with a new saline interface. In this video, we show the efficacy of this new strategy to optimize the presealing effect while using a small-tip knife ( Video 1 ).

A new technique, flushing during coagulation of the vessel, is used to improve the underwater presealing effect when using a small-tip knife.Video 1

At first, the formation of sparks in an underwater setting indicates an undesired cutting effect without proper coagulation of the vessel ( Fig. 1 **a** ). Microbubbles may be observed around the tip of the knife. In the second part of the video, continuous flushing was performed by a second operator through the waterjet channel of the J-type knife (ClearCut Knife J-type, 1.5 mm; Finemedix, Daegu). This resulted in the disappearance of high voltage current-related sparks, and the desired presealing effect on the vessel ( Fig. 1 **b** ). Of note, no microbubbles were observed. Finally, when flushing was stopped, this was associated with a return of spark formation, with the desired cutting of the presealed vessel then achieved ( Fig. 1 **c** ).

**Fig. 1:**
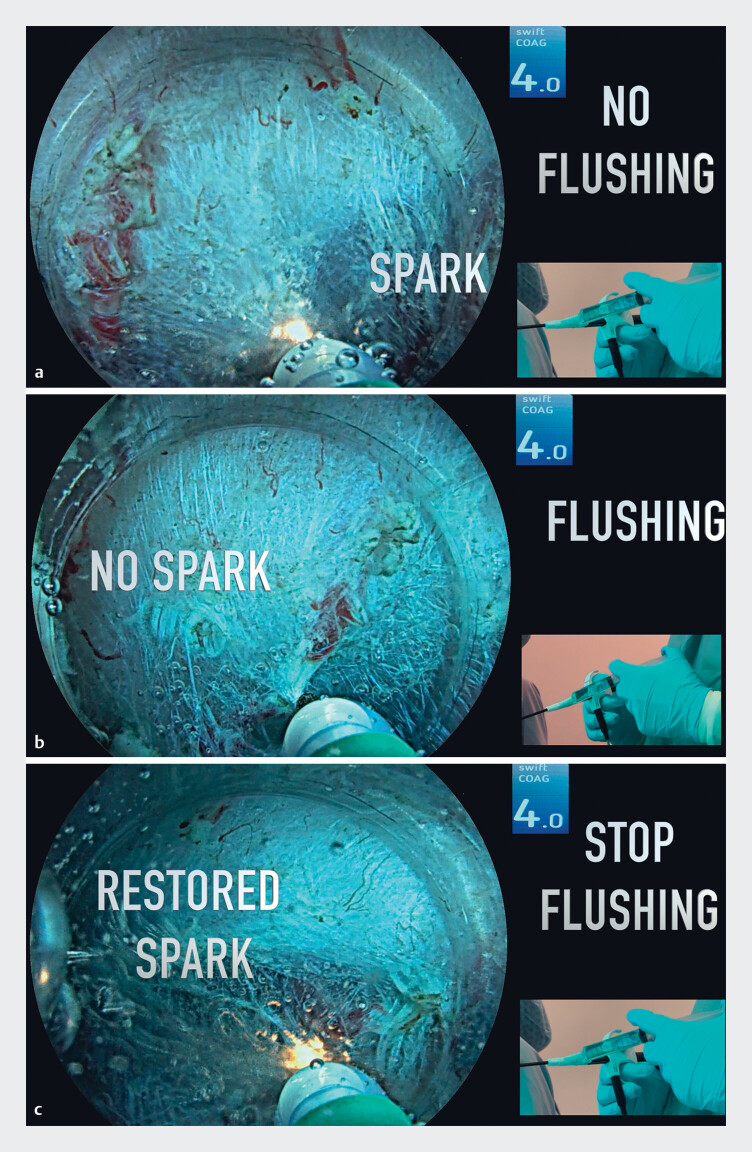
Endoscopic images showing: **a** a small spark formed at the tip of the knife when flushing is not being performed; **b** the presealing effect being achieved without spark formation while the area is being flushed; **c** restoration of spark formation when the flushing is stopped.

In conclusion, flushing assures the feasibility of presealing in underwater third-space endoscopy irrespective of the size of the knife. Familiarity with this technique is critical to prevent undesired intraprocedural bleeding in underwater third-space endoscopy.

Endoscopy_UCTN_Code_TTT_1AQ_2AD_3AD

Citation Format

Endoscopy 2025; 57: E213–E214. DOI: 10.1055/a-2528-6414

## Notes

Endoscopy E-Videos https://eref.thieme.de/e-videos
*E-Videos* is an open access online section of the journal *Endoscopy* , reporting on interesting cases and new techniques in gastroenterological endoscopy. All papers include a high-quality video and are published with a Creative Commons CC-BY license. Endoscopy E-Videos qualify for HINARI discounts and waivers and eligibility is automatically checked during the submission process. We grant 100% waivers to articles whose corresponding authors are based in Group A countries and 50% waivers to those who are based in Group B countries as classified by Research4Life (see: https://www.research4life.org/access/eligibility/ ). This section has its own submission website at https://mc.manuscriptcentral.com/e-videos .
